# Structural brain imaging studies offer clues about the effects of the shared genetic etiology among neuropsychiatric disorders

**DOI:** 10.1038/s41380-020-01002-z

**Published:** 2021-01-17

**Authors:** Nevena V. Radonjić, Jonathan L. Hess, Paula Rovira, Ole Andreassen, Jan K. Buitelaar, Christopher R. K. Ching, Barbara Franke, Martine Hoogman, Neda Jahanshad, Carrie McDonald, Lianne Schmaal, Sanjay M. Sisodiya, Dan J. Stein, Odile A. van den Heuvel, Theo G. M. van Erp, Daan van Rooij, Dick J. Veltman, Paul Thompson, Stephen V. Faraone

**Affiliations:** 1 Department of Psychiatry, SUNY Upstate Medical University, Syracuse, NY USA; 2 Departments of Psychiatry and of Neuroscience and Physiology, SUNY Upstate Medical University, Syracuse, NY USA; 3 Psychiatric Genetics Unit, Group of Psychiatry, Mental Health and Addiction, Vall d’Hebron Research Institute (VHIR), Universitat Autònoma de Barcelona, Barcelona, Spain; 4 Department of Psychiatry, Hospital Universitari Vall d’Hebron, Barcelona, Spain; 5 NORMENT—Institute of Clinical Medicine, Division of Mental Health and Addiction, Oslo University Hospital, University of Oslo, Oslo, Norway; 6 Radboudumc, Radboud University Medical Center, Nijmegen, The Netherlands; 7 Donders Institute for Brain, Cognition and Behaviour, Radboud University Medical Center, Nijmegen, The Netherlands; 8 Department of Cognitive Neuroscience, Radboud University Medical Center, Nijmegen, The Netherlands; 9 Imaging Genetics Center, USC Mark and Mary Stevens Neuroimaging and Informatics Institute, Keck School of Medicine of the University of Southern California, Marina Del Rey, CA USA; 10 Department of Human Genetics, Radboud University Medical Center, Nijmegen, The Netherlands; 11 Department of Psychiatry, Radboud University Medical Center, Nijmegen, The Netherlands; 12 Imaging Genetics Center, Department of Neurology and Biomedical Engineering, USC Mark and Mary Stevens Neuroimaging and Informatics Institute, Keck School of Medicine of USC, University of Southern California, Marina Del Rey, CA USA; 13 Department of Psychiatry, Center for Multimodal Imaging and Genetics (CMIG), University of California, San Diego, CA USA; 14 Centre for Youth Mental Health, The University of Melbourne, Parkville, VIC Australia; 15 Orygen, The National Centre of Excellence for Youth Mental Health, Parkville, VIC Australia; 16 UCL Queen Square Institute of Neurology, Department of Clinical and Experimental Epilepsy, University College London, London, UK; 17 Chalfont Centre for Epilepsy, Epilepsy Society, Bucks, UK; 18 SA MRC Unit on Risk & Resilience in Mental Disorders, Department of Psychiatry & Neuroscience Institute, University of Cape Town, Cape Town, South Africa; 19 Department of Psychiatry and Department of Anatomy & Neurosciences, Amsterdam UMC/VUmc, Amsterdam, The Netherlands; 20 Clinical Translational Neuroscience Laboratory, Department of Psychiatry and Human Behavior, University of California Irvine, Irvine, CA USA; 21 Center for the Neurobiology of Learning and Memory, University of California Irvine, Irvine, CA USA; 22 Donders Centre for Cognitive Neuroimaging, Radboud University Medical Center, Nijmegen, The Netherlands; 23 Neuro Imaging Institute for Neuroimaging and Informatics, Keck School of Medicine of the University of Southern California, Marina Del Rey, CA USA

**Keywords:** Genetics, Diseases

## Abstract

Genomewide association studies have found significant genetic correlations among many neuropsychiatric disorders. In contrast, we know much less about the degree to which structural brain alterations are similar among disorders and, if so, the degree to which such similarities have a genetic etiology. From the Enhancing Neuroimaging Genetics through Meta-Analysis (ENIGMA) consortium, we acquired standardized mean differences (SMDs) in regional brain volume and cortical thickness between cases and controls. We had data on 41 brain regions for: attention-deficit/hyperactivity disorder (ADHD), autism spectrum disorder (ASD), bipolar disorder (BD), epilepsy, major depressive disorder (MDD), obsessive compulsive disorder (OCD), and schizophrenia (SCZ). These data had been derived from 24,360 patients and 37,425 controls. The SMDs were significantly correlated between SCZ and BD, OCD, MDD, and ASD. MDD was positively correlated with BD and OCD. BD was positively correlated with OCD and negatively correlated with ADHD. These pairwise correlations among disorders were correlated with the corresponding pairwise correlations among disorders derived from genomewide association studies (*r* = 0.494). Our results show substantial similarities in sMRI phenotypes among neuropsychiatric disorders and suggest that these similarities are accounted for, in part, by corresponding similarities in common genetic variant architectures.

## Introduction

Neuropsychiatric disorders have substantial heritability, as shown by many studies of twins and families [1]. Genomewide association studies (GWAS) have shown that common genetic variants account for some of this heritability, and that some of this heritability is shared across neuropsychiatric disorders [2–5]. The genetic overlap across disorders may partly explain why these disorders tend to co-occur with one another in both clinical and community samples [6].

Subcortical brain volumes and cortical thickness/surface area dynamically change from early development through adulthood and old age. A study of the Enhancing Neuroimaging Genetics through Meta-Analysis (ENIGMA) Plasticity Working Group reported that changes in structural magnetic resonance imaging (sMRI) phenotypes have heritabilities ranging from 5% for pallidum to 42% for cerebellar gray matter [7]. Heritability estimates of change rates were age-related and generally higher in adults than in children, probably due to an increasing influence of genetic factors with age [7]. However, it appears that later in adulthood heritability decreases most likely due to cumulative effect of environmental influences over the lifespan [8]. ENIGMA sMRI studies of different psychiatric and neurological disorders further characterized MRI-derived phenotypes that can be used to assess heritability (reviewed in [9]).

ENIGMA has also reported significant case vs. control differences in sMRI phenotypes for: attention-deficit/hyperactivity disorder (ADHD) [10, 11], autism spectrum disorder (ASD) [12], bipolar disorder (BD) [13, 14], common epilepsy syndromes [15], major depressive disorder (MDD) [16, 17], obsessive compulsive disorder (OCD) [18, 19], and schizophrenia (SCZ) [20, 21]. Here we estimate the degree of similarity in sMRI phenotypes among these disorders and evaluate whether these similarities are influenced by corresponding similarities in common genetic variant architectures.

## Methods

### Collection of structural neuroimaging summary statistics

Summary statistics from ENIGMA structural neuroimaging studies were collected from 12 multisite analyses published by the ENIGMA Consortium for the following neuropsychiatric disorders: ADHD [10, 11], ASD [12], BD [13, 14], epilepsy [15], MDD [16, 17], OCD [18, 19], and SCZ [20, 21]. Prior to computing the summary statistics, the regional brain volumes had been segmented with a common ENIGMA protocol using FreeSurfer software. Each site performed these segmentations on their raw data. In addition, quality control protocols provided by ENIGMA were run at each site. Details are at: http://enigma.ini.usc.edu/protocols/imaging-protocols.

The ADHD and ASD samples comprised both youth and adults. The other samples comprised adults only. The ethnicity of the patients was not available for all participants. The “epilepsy” cohort comprised temporal lobe epilepsy, genetic generalized epilepsy, and extra temporal epilepsy. We analyzed 7 subcortical and 34 cortical regions (total of 41 brain regions; the mean of left and right structures) that were included in the above specified ENIGMA studies. We extracted the covariate-adjusted Cohen’s *d* standardized mean differences (SMDs) denoting the case versus unaffected comparison subject differences in subcortical volume and cortical thickness/surface area measures. The covariates used in these studies adjusted SMDs for several covariates as indicated in Supplementary Table 1.

### Collection of GWAS results among neuropsychiatric disorders

Publicly available summary statistics from GWAS were downloaded from the Psychiatric Genomics Consortium (PCG) website (https://www.med.unc.edu/pgc/results-and-downloads/) with the exception of GWAS results for MDD coming from an online resource hosted by the University of Edinburgh (10.7488/ds/2458) and of GWAS results for epilepsy coming from the online Epilepsy Genetic Association Database (epiGAD) (http://www.epigad.org/gwas_ilae2018_16loci.html). Presented in Supplementary Table 2 are the numbers of affected cases and unaffected control participants included in each GWAS. Note, the full meta-analysis GWAS of MDD that included data from 23andMe was not available for public release, thus we used the meta-analysis that combined results from the PGC cohorts and UK Biobank.

### Genetic and sMRI phenotype correlations among neuropsychiatric disorders

Linkage disequilibrium (LD)-score regression, a popular approach designed to analyze summary statistics from GWAS, was used to quantify the amount of shared genetic heritability, or genetic correlation (*r*_g_), existing between pairs of neuropsychiatric disorders, considering HapMap3 LD-scores [22]. For these analyses, the largest and latest GWAS available for each neuropsychiatric disorder was selected and filtered to exclude markers with INFO < 0.90 or within the MHC region (hg19:chr6:25–35 Mb) (Supplementary Table 1). GWAS summary statistics were merged with the HapMap3 reference panel (hg37 build), wherein variants with a MAF ≥ 5% in the HapMap3 dataset were retained, prior to computing (co)heritability estimates.

To derive an estimate of the degree to which sMRI phenotypes were similar among disorders, we computed pairwise Spearman’s rank correlation between the Cohen’s d SMDs for each pair of disorders. We then used Pearson’s correlation to estimate, whether the genetic correlations for each disorder covaried with the sMRI phenotype correlations. We used a traditional permutation framework to generate a null distribution of sMRI phenotype correlations by randomly shuffling Cohen’s *d* values 10,000 times for each pair of disorders, then recalculating sMRI correlations from the shuffled sets. From the null distributions, we derived an empirical permutation *p* value for each sMRI phenotype correlation. However, a reliable *p* value could not be calculated due to nonindependence between pairwise caused by sample overlap between imaging studies. Adjustments for sample overlap would be possible with individual-level data, but the present study only had access to summary statistics. In a leave-one-out analysis, we iteratively excluded one pair of disorder correlations from the set and recalculated Spearman’s correlation coefficients to determine whether correlations were driven by any pair of disorders. Binomial sign tests were used to determine whether the number of disorders showing the same direction of effect in the sMRI phenotypes was greater than expected by chance (null probability of 50%). Per brain region, we performed Cochran’s *Q* test implemented in the *R* package *metafor* (v.2.1–0) to determine whether variability among Cohen’s d values was greater than expected by chance. All statistical analyses were performed with *R* version 3.5.2 (R Core Team, 2018). We adjusted for repeated correlation tests using the Bonferroni procedure. Correlations showing a Bonferroni-adjusted *p* < 0.05 were considered significant (threshold *p* = 0.00227).

## Results

Sample demographics for the twelve studies by the ENIGMA Consortium on structural brain abnormalities in neuropsychiatric disorders are presented in Table 1.

**Table 1:** Sample demographics for the twelve studies by the ENIGMA Consortium into structural brain alterations in neuropsychiatric disorders.

Disorder	MRI measure	Cases (*n*)	Controls (*n*)	Total *n*	Sites	Weighted mean age (cases)	Weighted mean age (controls)	References
ADHD	Cortical thickness	2246	1934	4180	36	19.2	18.1	[2, 28]
	Surface area	2246	1934	4180	36	19.2	18.1	
	Subcortical volume	1713	1529	3242	23	18.6		
ASD	Cortical thickness	1571	1651	3222	49	15.4		[18]
	Surface area							
	Subcortical volume							
BD	Cortical thickness	1837	2582	4419	28	38.4^a^	35.6^a^	[19, 30]
	Surface area	1820	2582	4402	28	38.4^a^	35.6^a^	
	Subcortical volume	1710	2594	4304	20	40.1^a^	36.5^a^	
Epilepsy	Cortical thickness	2149	1727	3876	24	34.4	33.3	[7]
	Surface area							
	Subcortical volume							
MDD	Cortical thickness	1911	7663	9574	20	44.8^a^	54.6^a^	[6, 22]
	Surface area	1902	7658	9560	20	44.8^a^	54.6^a^	
	Subcortical volume	1728	7199	8927	15	43.3^a^	56^a^	
OCD	Cortical thickness	1498	1435	2933	27	32.1	30.5	[26, 41]
	Surface area	1497	1433	2930	27	32.1	30.5	
	Subcortical volume	1495	1472	2967	25	32.0	30.6	
SCZ	Cortical thickness	4474	5098	9572	39	32.3^a^	34.5^a^	[27, 34]
	Surface area	4434	5073	9507	39	32.3^a^	34.5^a^	
	Subcortical volume	2028	2540	4568	15	34.0^a^	31.0^a^	

^a^Weighted mean not provided in paper; computed from descriptive statistics.

### Case–control differences in subcortical volume and cortical surface area and thickness within neuropsychiatric disorders

**Fig. 1:**
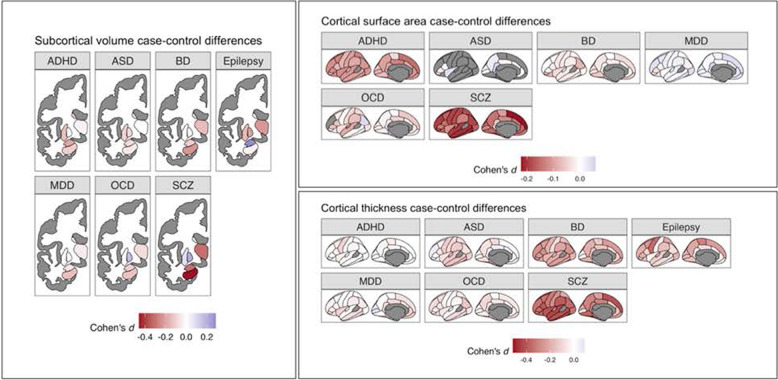
A brain imaging plot showing standardized mean differences (Cohen’s *d*) measuring case–control differences in subcortical volumes and cortical thickness for seven neuropsychiatric disorders. Results were obtained from ENIGMA working group publications. Negative values for Cohen’s *d* indicate smaller sizes of brain regions in cases versus unaffected comparisons. Note: ADHD attention-deficit/hyperactivity disorder, ASD autism spectrum disorder, BD bipolar disorder, MDD major depressive disorder, OCD obsessive compulsive disorder, SCZ schizophrenia.

Figure 1 presents an anatomical graph of the standardized effect sizes (Cohen’s *d*) measuring alterations in subcortical volume, cortical surface area and cortical thickness for 41 brain regions within seven neuropsychiatric disorders—ADHD, ASD, OCD, epilepsy, MDD, BD, and SCZ. These have been reported on prior publications. The variation in color from blue to red illustrates the phenomenon of SBRV, with some regions showing significant reductions (blue) in volume/thickness/surface areas and others not being affected. As indicated by the blueness of the cells, the most prominent reductions were seen for SCZ (mean Cohen’s d across all regions = −0.22, SE = 0.014), epilepsy (mean Cohen’s *d* = −0.12, SE = 0.017) and BD (mean Cohen’s *d* = −0.097, SE = 0.011). The smallest changes were observed for MDD (mean Cohen’s *d* = −0.018, SE = 0.006). All regions except for the caudate and putamen exhibited significant differences in the magnitude of Cohen’s d across disorders (Cochran’s Q *p* values = 0.012–2.8 × 10^−32^). Eighteen sMRI phenotypes exhibited homogeneity with respect to sign of Cohen’s *d* across each of the neuropsychiatric disorders evaluated (binomial sign test *p* values < 0.05): cortical thicknesses for caudal middle frontal gyrus, entorhinal cortex, fusiform gyrus, inferior temporal gyrus, insula, lateral orbitofrontal cortex, lingual gyrus, middle temporal gyrus, paracentral lobule, parahippocampal gyrus, pars opercularis of inferior temporal gyrus, precentral gyrus, precuneus, rostral anterior cingulate cortex, and supramarginal gyrus; subcortical volume for the hippocampus; and surface area for middle temporal gyrus, pars triangularis of inferior temporal gyrus, and pericalcarine cortex. For sMRI phenotypes for 39 regions of interest varying degrees of heterogeneity were noted in terms of discrepancy of signs of Cohen’s *d*. For example, individuals with ASD showed a slightly thicker cortex in the rostral middle frontal gyrus, individuals with ADHD showed no difference, and all other disorders showed a thinner cortex in this region compared to controls.

### sMRI phenotype correlations among neuropsychiatric disorders

**Fig. 2:**
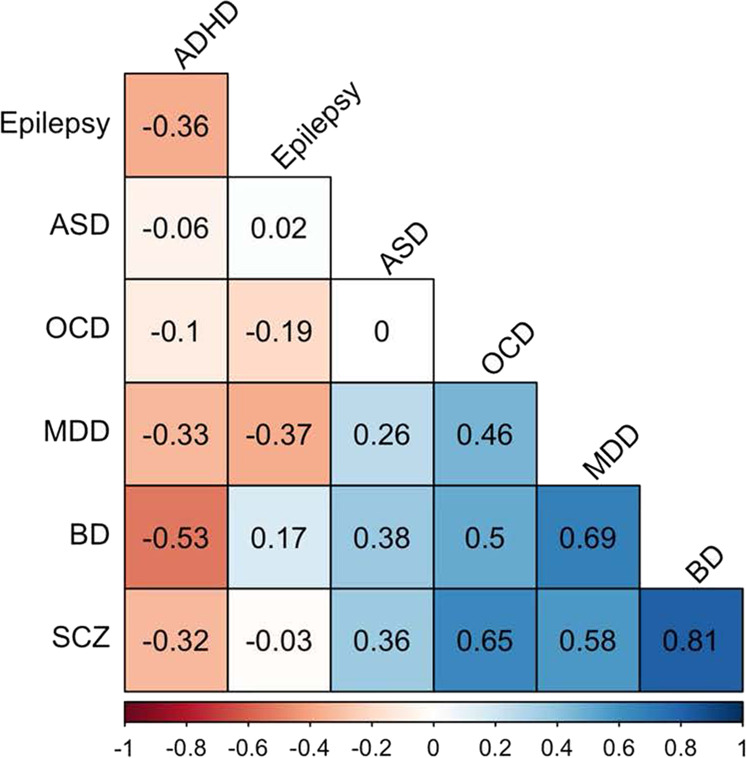
A heatmap of the cross-disorder pairwise sMRI correlations between seven neuropsychiatric disorders examined in this study. Colors in the plot correspond to the magnitude of the Pearson’s *r* coefficients, which are provided in each tile. Note: ADHD attention-deficit/hyperactivity disorder, ASD autism spectrum disorder, BD bipolar disorder, MDD major depressive disorder, OCD obsessive compulsive disorder, SCZ schizophrenia.

For each pair of disorders, we computed the Pearson correlation between their sMRI phenotypes listed in Fig. 1. These are listed in Table 2 (and visualized in Fig. 2), sorted by the magnitude of the correlation. The *p* values reported in Table 2 are potentially downwardly biased due to inability to properly adjust for spatial coherence of nearby brain regions. Traditional permutation *p* values are provided as a column in Table 2, which attempts to correct for potential biases due to spatial coherence. However, we were restricted from using a spatial permutation framework to generate a null distribution of correlations, because we are jointly analyzing two cortical maps (cortical thickness and surface area) that are fully overlapped. The highest positive correlation was between SCZ and BD (*r* = 0.81, df = 73, *p* < 1.3 × 10^−18^, Bonferroni *p* = 2.38 × 10^−17^). There were a few additional nominally significant negative correlations, which did not survive multiple testing correction: MDD and epilepsy (*r* = −0.37, *p* = 0.02), MDD and ADHD (*r* = −0.33, *p* = 0.004), SCZ and ADHD (*r* = −0.32, *p* = 0.005), ADHD and epilepsy (*r* = −0.36, *p* = 0.02), and a positive correlation between MDD and ASD (*r* = 0.26, *p* = 0.02).

**Table 2:** Cross-disorder structural MRI phenotype correlations (ordered from smallest to largest *p* value) based on Cohen’s *d* values obtained from the ENIGMA Project.

Disorder 1	Disorder 2	sMRI correlation Pearson’s *r*	df	se	*p* value	Boferroni adjusted *p* value	Permutation *p* value
BD	SCZ	0.81	73	0.068	1.13E−18	2.38E−17	<1E10−4
BD	MDD	0.69	73	0.085	1.21E−11	2.55E−10	<1E10−4
OCD	SCZ	0.65	72	0.09	5.53E−10	1.16E−08	<1E10−4
MDD	SCZ	0.58	73	0.095	5.55E−08	1.17E−06	<1E10−4
ADHD	BD	−0.53	73	0.099	1.18E−06	2.48E−05	<1E10−4
BD	OCD	0.5	72	0.102	4.74E−06	9.95E−05	<1E10−4
MDD	OCD	0.46	72	0.104	3.28E−05	6.89E−04	<1E10−4
ASD	BD	0.38	73	0.108	8.98E−04	0.02	<1E10−4
ASD	SCZ	0.36	73	0.109	1.35E−03	0.03	0.0176
ADHD	MDD	−0.33	73	0.111	4.27E−03	0.09	0.019
ADHD	SCZ	−0.32	73	0.111	4.63E−03	0.1	0.0014
Epilepsy	MDD	−0.37	39	0.149	0.02	0.38	0.0056
ADHD	Epilepsy	−0.36	39	0.149	0.02	0.41	0.004
ASD	MDD	0.26	73	0.113	0.02	0.46	0.024
Epilepsy	OCD	−0.19	39	0.157	0.23	1	0.22
BD	Epilepsy	0.17	39	0.158	0.3	1	0.3
ADHD	OCD	−0.1	72	0.117	0.39	1	0.39
ADHD	ASD	−0.06	73	0.117	0.6	1	0.6
Epilepsy	SCZ	−0.03	39	0.16	0.86	1	0.85
ASD	Epilepsy	0.02	39	0.16	0.91	1	0.91
ASD	OCD	0	72	0.118	0.97	1	0.97

### Correlation of shared genetic heritability with brain structural correlation

**Fig. 3:**
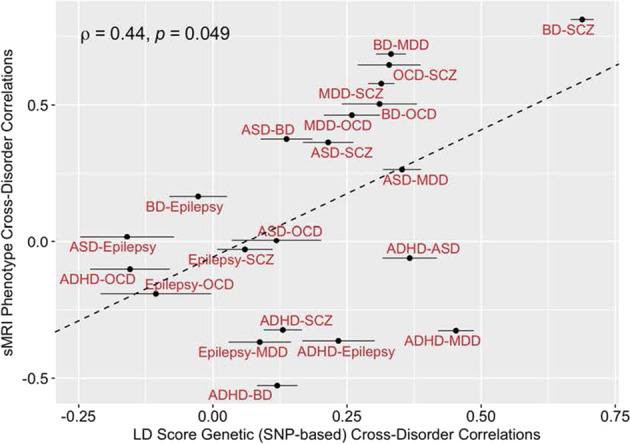
Scatter plot showing the correlation of correlations. Genetic correlations (rg) computed by LD-score regression are on the horizontal axis (with standard error bars), with correlations of Cohen’s *d* values displayed on the vertical axis. Each dot is color-coded according to the pairwise disorder correlations that were computed. The dotted line represents the best-fit regression line. The Spearman’s rho (*ρ*) and *p* value are provided at the top-left corner of the panel. Note: ADHD attention-deficit/hyperactivity disorder, ASD autism spectrum disorder, BD bipolar disorder, MDD major depressive disorder, OCD obsessive compulsive disorder, SCZ schizophrenia.

Figure 3 shows the pairwise correlations of sMRI phenotypes and genetic overlap across each pair of neuropsychiatric disorders. The LD-score cross-disorder genetic correlations are positively correlated with the sMRI phenotype cross-disorder correlations (Spearman’s *ρ* = 0.44, *p* = 0.049). Leave-one-out sensitivity analyses confirmed that the direction of the correlation was positive and remained moderate in magnitude despite removal of individual pairs of disorders from the correlation test (range of Spearman’s *ρ* = 0.35–0.58), except for removing SCZ/BD (Spearman’s *ρ* = 0.35). SCZ and BD showed the highest degree of concordance with respect to genetic and sMRI phenotype correlations.

## Discussion

Our analysis of summary statistics from the ENIGMA ADHD, ASD, BD, MDD, OCD, SCZ, and epilepsy Working Groups and the predominantly PGC case–control GWAS identified two novel findings. First, we found substantial correlations for some disorders in the pattern of sMRI case–control differences across subcortical and cortical regions in line with recently published study of [23]. Second, these cross-disorder correlations in SBRV could partly be explained by the genetic correlations reported for these disorders from GWAS [3].

The cross-disorder correlations in SBRV are intriguing because, like cross-disorder genetic correlations, they suggest that these disorders, to varying degrees, share aspects of their etiology and pathophysiology. Any interpretation of the cross-disorder sMRI correlations must keep in mind that, for all disorders, the case–control differences in sMRI measures are small (Fig. 1). The largest Cohen’s *d* values are only −0.5 for SCZ [20, 21], −0.4 for epilepsy [15], −0.3 for BD [13, 14], −0.2 for ADHD [10, 11] and ASDs [12], and −0.1 for MDD [16, 17] and OCD [18, 19]. These small case–control differences are consistent with results from GWAS and environmental risk studies, which speaks to the fact that the effects of common risk factors are, with some rare exceptions, individually small. Although it is conceivable that these small risks could accumulate to create a more dramatic pathophysiology in the brain, the ENIGMA data show that this is not the case for sMRI measures. Consistent with this finding, interindividual differences in neuroimaging account for only a small amount of the variance in symptom expression or behavioral measures of symptomatic or behavioral variance [24].

The most prominent case–control differences in cortical thickness/surface area and subcortical volumes were observed for SCZ [20, 21] and BD [13, 14]. These disorders also had the highest sMRI phenotype correlations and both also showed strong sMRI phenotype correlations with MDD [16, 17] and OCD [18, 19]. As Fig. 2 shows, these disorders clustered together in the three-dimensional configuration required to capture cross-disorder sMRI phenotype similarity. The high sMRI correlation between SCZ and BD is consistent with prior reports of sMRI similarities between the two disorders [25]. Moreover, a large body of literature reports substantial etiologic overlap between the two disorders [26–30]. Because of such data, the SCZ and BD have been described as sharing a continuum of etiology leading to psychotic [31], neurophysiological [31] and neurocognitive [32] symptoms. The ENPACT study [33] showed shared fronto–temporo–occipital gray matter volume deficits in the right hemisphere of two disorders. A systematic review of associations between functional MRI activity and polygenic risk for SCZ and BD [26] reported that genetic load for these disorders affects task-related recruitment of predominantly frontal lobe brain regions.

Many studies have reported that OCD can be a comorbid diagnosis with SCZ or that patients with SCZ can have OCD symptoms [34–41]. Presented findings of a significant overlap in sMRI phenotypes along with the known SCZ/OCD genetic correlations suggests that more work should examine shared pathophysiologic features between these disorders and should assess the degree to which confounds, such as medication status or chronicity, might explain these results.

The sMRI phenotype correlations mirror, to some extent, the cross-disorder correlations from GWAS. Figure 3 shows a modest, yet distinct, linear correlation between the sMRI phenotype and genetic correlations. In the upper right-hand section of the plot, we see disorders having high genetic and high sMRI correlations. These are SCZ/BD, SZ/MDD, BD/MDD, OCD/BD, and OCD/MDD. The inclusion of MDD in this group is notable given that it is part of the bipolar diagnosis and often occurs comorbid with other disorders. MDD also has a high genetic correlation with ADHD but a negative sMRI correlation, which makes that pair an outlier in Fig. 3.

In the lower left region of Fig. 3, we see disorders with low genetic and low sMRI correlations. These involve correlations of epilepsy, and correlations of ADHD with all disorders except ASDs and MDD, although the latter is somewhat of an outlier. ASDs tend to have both modest genetic correlations and modest sMRI correlations with most other disorders and, hence, populates the middle range of the figure. Like the sMRI correlations among disorders, all genetic correlations with epilepsy are low, which is consistent with the low genetic correlation between neurological and psychiatric disorders as reported by [2].

The finding that SBRV correlations are correlated with genetic correlations suggests that future studies of SBRV should consider genetic sources of etiology. Yet, because only about 24% of the variance in the SBRV correlations can be accounted for by the genetic correlations, environmental sources of etiology and disease-specific genetic contributions must also be considered. These include shared confounders, such as chronicity and medication exposure, along with shared etiologic events such as birth complications or exposure to toxins in utero. Our prior studies of SBRV in ADHD implicated the regulation of genes in apoptosis, autophagy and neurodevelopment pathways in ADHD [42, 43]. Neurodevelopmental pathways had also been implicated in the cross-disorder analysis of the PCG [3], which suggests that cross-disorder similarities in these pathways may account for cross-disorder similarities in SBRV.

Although we used data derived from very large samples (ENIGMA, iPSYCH, and the PGC), several limitations moderate the strength of our conclusions. We inherit all the limitations of the constituent studies, but are further limited because we analyzed summary statistics, not the original data, which would require the sharing of individual subject level data, an ongoing effort among the ENIGMA disorder working groups. Thus, we cannot determine whether the possible use of controls shared among studies affected our results. It is also possible that some research participants were included in the genetic and sMRI data sets for the same disorder. The *p* value obtained by our Spearman’s correlation test of cross-disorder sMRI and genetic correlations may be inaccurate due to spatial autocorrelation among sMRI Cohen’s *d* estimates, which can downwardly bias standard errors and lead to deflated *p* values. Considering we are not able to completely address with autocorrelation among brain regions using summary statistics alone, the *p* value from our primary analysis (presented in Fig. 3) should be interpreted with caution. Another problem is that we could not address effects of medications or chronicity on brain structure. Furthermore, for some of the disorders, we could use youth and adult data, whereas for others only adult effect data were used. Because findings can differ substantially depending on the age range of the samples included (e.g., [10, 11, 18, 19], this might have influenced our findings. For these reasons, analyses of participant level data will be needed to address these issues to draw stronger and more detailed conclusions. We also did not have any longitudinal data available, which limits the ability to test hypotheses about shared and unique developmental trajectories among disorders.

Despite these limitations, we have documented cross-disorder correlations in SBRV as assessed by sMRI. These cross-disorder SBRV correlations are positively associated with the disorders’ corresponding cross-disorder genetic correlations. This finding is a novel contribution worthy of further study that contributes to novel body of literature focused on cross-level correspondence of genetic and neuroimaging presentations of different psychiatric disorders [44–49]. Our work supports conclusions from previous GWAS studies suggesting a partially shared etiology and pathophysiology among many disorders [2, 50]. Disorders like SCZ and BD or ADHD and ASD, which are distinct in the diagnostic nomenclature, show significant overlap in etiology and pathophysiology. Further studies are needed to discern why brain regions are selectively affected by the risk factors that cause sMRI abnormalities [42, 43] and why these effects are correlated across disorders. Such studies may give insights into new treatment targets.

## Supplementary information

Supplementary Materials

## Data Availability

*URLs for GWAS* SCZ from ckqny.scz2snpres.gz (https://www.med.unc.edu/pgc/results-and-downloads), ASD from iPSYCH-PGC_ASD_Nov2017.gz (https://www.med.unc.edu/pgc/results-and-downloads), OCD from PGC_OCD_Aug2017-20171122T182645Z-001.zip > ocd_aug2017.gz (https://www.med.unc.edu/pgc/results-and-downloads), ADHD from adhd_ul2017.gz (https://www.med.unc.edu/pgc/results-and-downloads), BD from daner_PGC_BIP32b_mds7a_0416a.gz (https://www.med.unc.edu/pgc/results-and-downloads), Epilepsy from all_epilepsy_METAL.gz (http://www.epigad.org/gwas_ilae2018_16loci.html), and MDD from PGC_UKB_depression_genome-wide.txt (10.7488/ds/2458).

## Code availability

Custom *R* scripts used to generate results in this study can be made available upon request.
