# Supplementary material for: Structural brain imaging studies offer clues about the effects of the shared genetic etiology among neuropsychiatric disorders
Source: Mol Psychiatry. 2021 Jan 17;26(6):2101–10. doi: 10.1038/s41380-020-01002-z (PMC8440178; doi:10.1038/s41380-020-01002-z)
Supplement: Supplementary file 1 — Supplementary Materials [file 41380_2020_1002_MOESM1_ESM.docx]

**Supplementary Table 1.** Covariate Adjustments Used by Each Study

| **Disorder** | **Brain measure** | **Covariates** |
| --- | --- | --- |
| Bipolar disorder | Cortical thickness and surface area | Age, sex, scan center, ICV (surface area only) |
| Bipolar disorder | Subcortical volume | Age, sex, ICV |
| Depression | Cortical thickness and surface area | Age, sex, scan center |
| Depression | Subcortical | Age, sex, scan center, ICV |
| Schizophrenia | Cortical thickness and surface area | Age, sex |
| Schizophrenia | Subcortical | Age, sex, ICV |
| OCD | Cortical thickness and surface area | Age, sex, scan center, ICV (surface area only) |
| OCD | Subcortical | Age, sex, scan center, ICV |
| ASD | Cortical thickness and surface area | Age, sex, scan center  (groups matched on ICV) |
| ASD | Subcortical | Age, sex, scan center (groups matched on ICV) |
| Epilepsy | Cortical thickness and surface area | Age, sex, ICV |
| Epilepsy | Subcortical | Age, sex, ICV |
| ADHD | Cortical thickness and surface area | Age, sex, scan center, ICV (surface area only) |
| ADHD | Subcortical | Age, sex, ICV |

**Supplementary Table 2.** Additional information pertaining to the genomewide association studies (GWASs)

| **Disorder** | **Number of cases** | **Number of controls** | **Estimated SNP-h^2^ (SE)** | **References** | **Link to dataset** |
| --- | --- | --- | --- | --- | --- |
| ADHD | 20,183 | 35,191 | 0.23 (0.014) | Demontis et al., 2019 (Nat Genet) | https://www.med.unc.edu/pgc/results-and-downloads |
| Autism spectrum disorder | 18,382 | 27,969 | 0.20 (0.016) | Grove et al., 2019  (Nat Genet) | https://www.med.unc.edu/pgc/results-and-downloads |
| Bipolar disorder | 20,352 | 31,358 | 0.35 (0.016) | Stahl et al., 2019 (Nat Genet) | https://www.med.unc.edu/pgc/results-and-downloads |
| Epilepsy | 15,212 | 29,677 | 0.11 (0.017) | [The International League Against Epilepsy Consortium on Complex Epilepsies](https://www.nature.com/articles/s41467-018-07524-z#group-1), 2018  (Nat Commun | http://www.epigad.org/gwas_ilae2018_16loci.html |
| Major depressive disorder | 170,756 | 329,4438 | 0.06 (0.0023) | Howard et al., 2019  (Nat Neurosci) | <http://dx.doi.org/10.7488/ds/2458> (MDD dataset excluding 23andMe) |
| Obsessive compulsive disorder | 2,688 | 7,037 | 0.33 (0.048) | [International Obsessive Compulsive Disorder Foundation Genetics Collaborative (IOCDF-GC) and OCD Collaborative Genetics Association Studies (OCGAS)](https://www.ncbi.nlm.nih.gov/pubmed/?term=International%20Obsessive%20Compulsive%20Disorder%20Foundation%20Genetics%20Collaborative%20(IOCDF-GC)%20and%20OCD%20Collaborative%20Genetics%20Association%20Studies%20(OCGAS)%5BCorporate%20Author%5D) 2018 (Mol Psychiatry) | https://www.med.unc.edu/pgc/results-and-downloads |
| Schizophrenia | 36,989 | 113,075 | 0.24 (0.009) | Ripke et al., 2014 (Nature) | https://www.med.unc.edu/pgc/results-and-downloads |
